# Overweight and obesity in type 1 diabetes is not associated with higher ghrelin concentrations

**DOI:** 10.1186/s13098-021-00699-4

**Published:** 2021-07-22

**Authors:** Behiye Özcan, Patric J. D. Delhanty, Martin Huisman, Jenny A. Visser, Sebastian J. Neggers, Aart Jan van der Lely

**Affiliations:** Departments of Internal Medicine, Erasmus MC, University Medical Center Rotterdam, PO Box 2040, 3000 CA Rotterdam, The Netherlands

**Keywords:** Ghrelin, Unacylated ghrelin, Desacylated ghrelin, diabetes type 1, Insulin, Body mass index

## Abstract

**Background:**

Several studies have demonstrated suppressed levels of acylated (AG) and unacylated ghrelin (UAG) in patients with type 2 diabetes. However, the role of these hormones in type 1 diabetes has not been extensively studied. This study assessed the relationship between AG and UAG levels and body composition in patients with type 1 diabetes.

**Methods:**

We selected eighteen patients with type 1 diabetes and divided them into two groups: non-obese (BMI < 25 kg/m^2^) and overweight (BMI ≥ 25 kg/m^2^). Demographics, parameters of body composition and serum parameters including AG and UAG, were assessed.

**Results:**

The patients with a BMI ≥ 25 kg/m^2^ were older and had a longer duration of diabetes. AG and UAG levels were not significantly different between non-obese and overweight groups (mean AG non-obese ± SD: 44.5 ± 29.4 pg/ml and mean UAG non-obese 42.4 ± 20.7 pg/ml vs mean AG overweight ± SD: 46.1 ± 29.6 pg/ml and mean UAG overweight 47.2 ± 18.2 pg/ml). AG/UAG ratios did not discriminate between these groups. There was a positive association of insuline dose/kg bodyweight with BMI (r^2^ = 0.45, p = 0.002).

**Conclusions:**

Surprisingly, unlike non-diabetics and in T2D, we did not observe a difference in plasma levels of AG and UAG between normal weight and overweight adult type 1 diabetics. However, we did observe a positive correlation between BMI and insuline dose/kg bodyweight, suggesting that exogenous insulin is more important than the ghrelin system in the development of obesity in type 1 diabetes.

## Background

Ghrelin is the natural ligand of the growth hormone secretagogue receptor type-1a (GHSR-1a) [1] and might play an important role in obesity and metabolic-related disorders [2–5]. Ghrelin expression and secretion are increased when energy balance is negative and it is known to be an independent predictor of insulin resistance. Fasting plasma ghrelin levels are also negatively correlated with BMI [6].

In obesogenic conditions, ghrelin levels are reduced, with a concomitant induction of chronic low-grade inflammation [5, 7]. Ghrelin is most known for its role in appetite regulation, acting directly on hypothalamic neurons responsible for feeding behavior [8]. As a result of the obesity pandemic, many studies report on the potential association between ghrelin levels and body composition. For example, patients with anorexia nervosa have elevated plasma ghrelin levels [9, 10] which decline with body weight gain. Furthermore, other studies suggest that ghrelin has positive trophic activity, protecting β-cells against damage in experimental models of type 1 diabetes [11, 12].

The function of the ghrelin axis has not been addressed extensively in type 1 diabetes. Moreover, data on plasma ghrelin levels in patients with type 1 diabetes are conflicting. For example, the onset of type 1 diabetes is known to be associated with decreased circulating ghrelin levels [13, 14]. In one study, ghrelin levels were reported as being low prior to insulin treatment and non-responsive to meal tests in children with newly diagnosed type 1 diabetes [15]. This has been confirmed in another study, which reported that total and acylated ghrelin (AG) concentrations were lower in children with type 1 diabetes [14]. On the other hand, another study observed higher total plasma ghrelin concentrations in type 1 diabetes patients, which declined by 29% after insulin treatment [16]. Murdolo et al. [17] have shown that in untreated type 1 diabetes patients post-prandial ghrelin levels are not suppressed, which may contribute to the hyperphagia that is often observed in these patients. Furthermore, the above mentioned studies measured only total ghrelin levels, and did not differentiate between the two forms found in the circulation, AG and unacylated ghrelin (UAG) [18]. Accordingly, in untreated streptozotocin (STZ)-induced diabetic rats, an animal model for type 1 diabetes, gastric and plasma ghrelin concentrations were reported to be increased [19–21]. Support for the concept of the contribution of the ghrelin signaling pathway to the development of STZ-induced diabetic hyperphagia has been provided by two studies analyzing ghrelin knockout [22] and ghrelin-receptor knockout mice [23].

Although patients with type 1 diabetes have traditionally been thought to have a lower BMI than patients with type 2 diabetes, recent research has shown otherwise [24, 25].

Because of the obesogenic effect of ghrelin and the fact that obesity in type 1 diabetes is frequently observed, we studied the relationship between ghrelin concentrations, BMI, metabolic control and insulin dosage.

## Methods

### Patients

We selected 18 patients with type 1 diabetes who were lean at diagnosis from our hospital out-patient clinic. After we had obtained written informed consent, all subjects underwent a physical examination and fasting serum samples were collected. Exclusion criteria included diabetes type 2, monogenetic forms of diabetes, systemic corticosteroid use < 60 days prior to screening, pregnancy or breast feeding, drug or alcohol dependence or abuse, and participation in another trial at the same time. The study was approved by the local human ethical review board. The patients were divided into two groups: patients with a BMI < 25 and patients with a BMI ≥ 25. We collected demographics, BMI, and measured several serum parameters after overnight fast, including AG and UAG concentrations. Four of them were on anti-hypertensive treatments (22%). Eight patients were known with diabetic retinopathy and six with diabetic neuropathy. Two patients have nephropathy, one had TIA and one patient had cardiovascular diseases. Ten of the patients were using continuous subcutaneous insulin infusion (56%).

Levels of lipids, HbA1c, anti-GAD65 and serum creatinine were measured in venous plasma. Urinary albumin and creatinine were measured in a sample of urine. Levels of HbA1c were measured by high-performance liquid chromatography.

This study was approved by the local human ethical review board (The Medical Ethics Review Committee, protocol number MEC-2015-602) with written informed consent obtained from each participant.

### Hormone measurements

#### Ghrelin measurements

For AG and UAG measurements blood was collected into 4 ml EDTA tubes (Breda, Netherlands; cat# 367899; 6 mL K2 EDTA). Immediately after sample collection, AEBSF (Roche, Netherlands) was added to the blood samples (final concentration of 2 mg/ml) to prevent des-acylation of AG [26, 27]. Tubes were carefully mixed by inversion and stored on water ice (0 °C) until centrifugation at 2500*g* at 4 °C for 5 min. Plasma samples were stored in 300 µL aliquots at − 80 °C until assayed for AG and UAG. Human AG and UAG were determined by a double-antibody sandwich technique using enzyme immunometric assay (EIA) kits obtained from Bertin Pharma (Montigny-le-Bretonneux, France; Easy-sampling kits, A05306 and A05319). After slowly thawing on water ice, all plasma samples were briefly cleared by centrifugation before transferring into the assay plates. All samples were analysed in duplicate (50 µL/well) according to the manufacturer’s protocol. Intra-assay coefficient of variation (%CV) is typically 2.7% CV for AG and 3.4%CV for UAG. Inter-assay %CV is 13.2%CV and 15.0% CV, for AG and UAG respectively (manufacturer’s suggested cut-off = 25%).

### Statistical analysis

Differences between groups were analyzed using the Mann–Whitney U test as an unpaired t test because of nonparametric data. Results of correlation analyses were expressed as Spearman's rank correlation coefficients (rho). p values < 0.05 (two-tailed) were considered statistically significant. Statistical analyses were done using GraphPad Prism version 6 for Windows (GraphPad Software, San Diego, Calif., USA).

## Results

Table 1 shows the characteristics of the 9 normal weight and 9 obese type 1 diabetes patients. The patients with a BMI ≥ 25 kg/m^2^ were older and had a longer duration of diabetes than the patients with a normal BMI. The mean HbA1c of the obese patients (7.6%, 60 mmol/mol) did not differ from the lean patients (7.4%, 57 mmol/mol). The proportion of smokers was the same in both groups.

**Table 1:** Baseline characteristics of non-obese and overweight patients with type 1 diabetes

	BMI < 25^a^	BMI ≥ 25^a^	P < 0.05
Patients (men)	9 (6)	9 (4)	NS
Mean BMI (kg/m^2^)	22.7 ± 1.8	30.12 ± 3,4	NS
Age (years)	41 ± 9	54 ± 15	NS
Age at diagnosis	24 ± 13	24 ± 14	NS
Duration of diabetes (years)	23 ± 18	28 ± 17	NS
Alcohol users (n)	9	7	NS
Smoking (n)	4	4	NS
Number of statin users	4	7	NS
Mean systolic RR (mmHg)	131 ± 16	149 ± 17	NS
Mean diastolic RR (mmHg)	75 ± 13	73 ± 11	NS
Creatinine (μmol/L)	75 ± 8	89 ± 21	NS
Triglyceride (mmol/L)	1.03 ± 0.51	1.38 ± 0.69	NS
Total cholesterol (mmol/L)	4.6 ± 0.7	4,6 ± 1.3	NS
Mean LDL-cholesterol (mmol/L)	2.6 ± 0.9	2.7 ± 1.0	NS
apo B (g/L)	0.8 ± 0.2	1.0 ± 0.4	NS
mean HbA1C (mmol/mol)	53.5 ± 6.5	60.3 ± 7.2	NS
Albumin/creatinine ratio (g/mol)	0.01 ± 0.00	0.02 ± 0.02	NS
Mean acylated ghrelin (pg/ml)	44.5 ± 29.4	46.1 ± 29.6	NS
Mean unacylated ghrelin (pg/ml)	42.4 ± 20.7	47.2 ± 18.2	NS
ratio AG/UAG	1.1 ± 0.6	1.0 ± 0.5	NS
mean insulin dose (range 20 to 100) units per day	36.6 ± 17.0	52.6 ± 27.2	P < 0.02
Positive anti-GAD65	4	6	NS

^a^Continuous variables are expressed as mean ± standard deviation

LDL cholesterol levels were lower (although not significant) in patients with a high BMI.

Patients with a BMI ≥ 25 kg/m^2^ tended to have higher levels of albuminuria.

Both AG and UAG levels did not differ between the two groups (mean ± SD: 46.1 ± 29.6 vs 47.2 ± 18.2 pg/ml respectively in the obese versus lean patients). Also, the AG/UAG ratios did not differ significantly. The number of patients positive for anti-GAD-65 antibodies was higher in the patients with a high BMI.

**Fig. 1:**
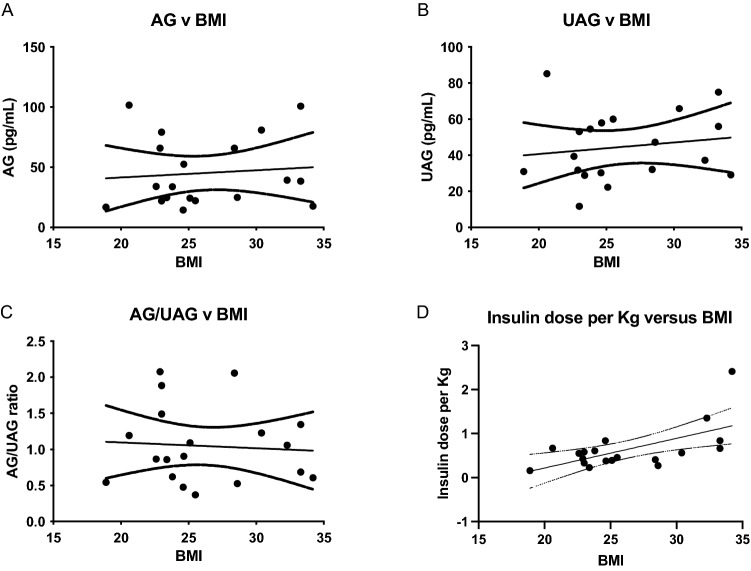
Correlations between BMI and AG (**A**), UAG (**B**), AG/UAG ratio (**C**) and insuline dose/kg bodyweight in IU/kg (**D**). *AG* acylated ghrelin in pg/ml, *UAG* unacylated ghrelin in pg/ml, *BMI* body mass index in kg/m^2^

BMI showed no correlation with either AG, UAG or AG/UAG ratio. Insuline dose/kg bodyweight was positively associated with BMI (r^2^ = 0.25, p = 0.02, Fig. 1A–D). In both groups, AG and UAG levels showed no correlation with HbA1c, insulin dose, or duration of the disease.

**Fig. 2:**
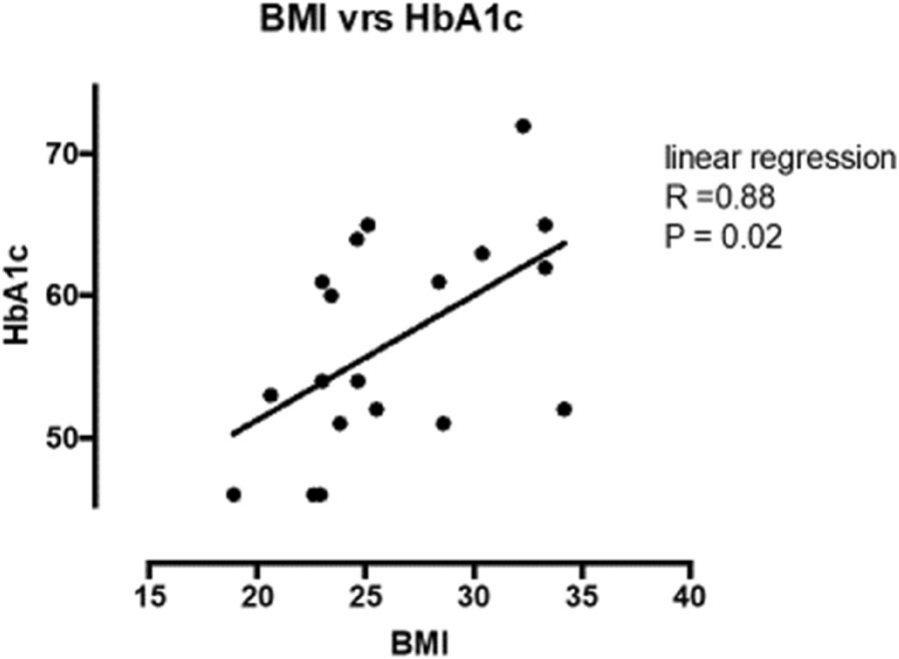
Linear regression analysis between BMI and HbA1c. HbA1c in mmol/mol and *BMI* body mass index in kg/m^2^

**Fig. 3:**
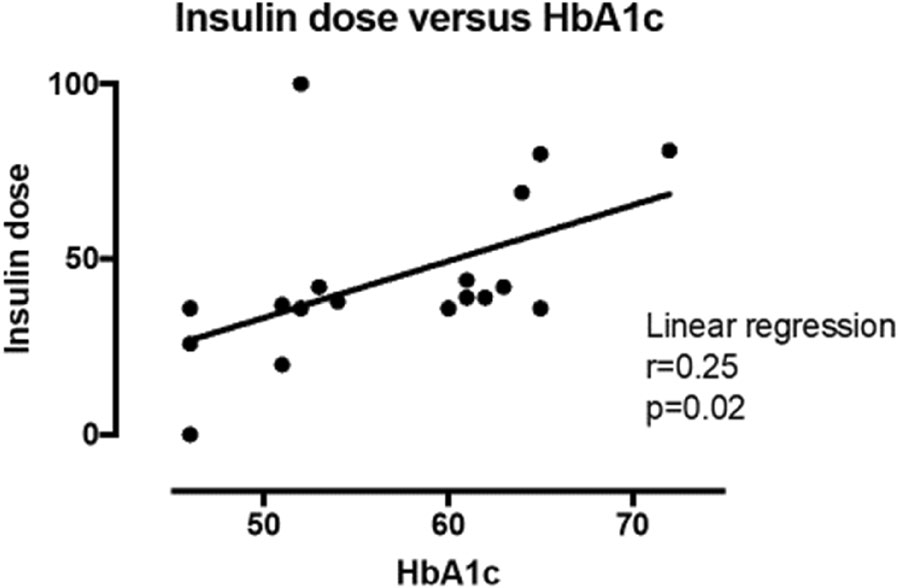
Linear regression analysis between daily insulin dose in IU and HbA1c in mmol/mol

Linear regression analysis revealed that HbA1c had a moderate positive association with BMI (Fig. 2). The relationship between insulin dose and HbA1c was weak (Fig. 3).

AG/UAG ratio was lower in the smoking type 1 patients. There was no association between smoking and the use of statins in these small groups of subjects.

## Discussion

We have investigated the plasma levels of AG and UAG in lean and obese patients with type 1 diabetes and found that obesity in type 1 diabetes appears to be more insulin mediated than by the ghrelin system. When we compared the plasma levels of AG and UAG between lean and obese patients with type 1 diabetes we found no differences between the absolute concentrations nor between the ratios of AG/UAG.

Noteworthy, enteroendocrine cells are distributed throughout the gastrointestinal tract producing a variety of peptides such as ghrelin and GLP-1. These two hormones are potently influenced each other in terms of feeding regulation. In healthy subjects ghrelin infusion demonstrates that ghrelin-induced GLP-1 reduces the effects of ghrelin to suppress β-cell function and worsen glucose tolerance [28].

Because of their type 1 diabetes, all our patients lack portal insulin signaling to the liver. Only a few studies have investigated incretins in people with type 1 diabetes. In an islet cell antibody positive group of people, disposed to develop type 1 diabetes, both fasting and postprandial plasma levels of GIP and GLP-1 were normal [29]. In 16 lean, primarily C-peptide negative type 1 diabetic patients with longstanding diabetes, fasting GLP-1 concentrations did not differ from those of normal subjects, whereas the GLP-1 secretion to a mixed meal was virtually absent [30]. In contrast to this, Vilsbøll and co-workers observed that the incremental response of GLP-1 and GIP following a meal did not differ between C-peptide negative type 1 diabetic patients and a glucose tolerant control group, but that the fasting level of intact GLP-1 tended to be lower in the type 1 diabetic subjects [31]. Interesting in respect to our observations, the authors speculated that their findings could be due to a negative feedback mechanism from exogenous insulin.

We also did not observe significant positive or negative correlations between BMI and either the levels of AG and UAG, or with the AG/AUG ratio. These findings indicate that in our small study population the ghrelin system appears not to be a major player in overweight or obesity in type 1 diabetes and that insulin treatment is likely a more important factor behind weight gain in these patients. In addition, the lack of correlation of serum AG and UAG levels with HbA1c and insulin dose suggests that ghrelin levels are not affected by these parameters.

Our findings contrast with those of Polkowska et al. who reported a negative correlation between BMI and the levels of AG in a subgroup of children with the longest diabetes duration (more than 10 years) [32]. UAG was not assessed in this study. Our findings also differ from a study of Ueno et al. [33], who analyzed the role of ghrelin in micro and macrovascular diabetic complications and glycemic control. Patients with type 1 diabetes and type 2 diabetes were classified into a lean, normal weight and an overweight group. Plasma ghrelin concentration were lower in diabetes patients with poor long-term glycemic control than in patients with a good long-term glycemic control. They also observed that obesity may influence ghrelin levels. The plasma ghrelin concentrations were lower in obese patients, which suggests that obesity may influence the regulation of ghrelin production. The mean HbA1c in their group was comparable to our study (56 and 57 mmol/mol).

Noteworthy however, is the fact that the methods of assaying ghrelin levels significantly differ between studies reported in the literature. In many previous studies, including the Ueno study [33], total ghrelin was measured using radioimmunoassays, which detect both full-length, and inactive fragments, of both ghrelin isoforms [33]. Other studies, unlike ours [26], did not stabilize samples with an esterase inhibitor to prevent deacylation of AG [32]. The stability of AG may have affected the AG concentrations. We have used AEBSF to stabilize ghrelin in blood samples from patients [26].

We studied the role of the ghrelin system in subjects without endogenous insulin production. Ghrelin however does play a role in the control of insulin secretion.

For example, pharmacological inhibition of ghrelin O-acyl-transferase (GOAT) in mice has been shown to improve glycemic control and stimulates the release of insulin [34]. Although, another study failed to confirm these findings [35], the GOAT-ghrelin systems does seem to play an essential role in preventing hypoglycemia during extreme episodes of calorie restriction [36].

Ghrelin inhibits insulin secretion via stimulation of the growth hormone secretagogue receptor 1a (GHSR1a), a process which also involves interaction with the somatostatin receptor subtype-5 [37]. As well as being a negative regulator of insulin secretion, ghrelin also seems to have protective effects on the β-cells [11, 12] despite evidence showing that levels of ghrelin decline with the onset of type 1 diabetes [13, 14].

Both in patients with type 2 and type 1 diabetes, the clinical course and metabolic control are affected by the degree of insulin resistance. For example, children with type 1 diabetes have higher levels of insulin resistance than their non- diabetic peers [38]. Published data suggest that it is the insulin resistance and not the obesity that causes suppression of ghrelin secretion. However, there is no agreement to whether or not lower levels of ghrelin are the result- or the cause of insulin resistance [6]. This may explain why the patients in our study who had the longest disease duration and the highest mean glycosylated hemoglobin tend to have the lowest concentrations of AG and UAG.

Previous reports have shown that amongst patients with type 2 diabetes, fasting plasma ghrelin concentrations are the lowest in obese patients and the highest in lean patients [39–41].

Although the role of AG and UAG has been investigated in obesity and type 2 diabetes, the exact regulation of their secretion in type 1 diabetes is currently unknown [13, 14, 16, 42].

Previous studies have also looked at the effect of statins on ghrelin. In rodents, statins in different dosages and different time points over the day had no significant effects on ghrelin levels [43]. In patients with type 2 diabetes, statins did not significantly change ghrelin levels after 12 months of treatment [44].

To our knowledge, no data are available in the literature on UAG levels in patients with long-term type 1 diabetes. The follow-up period in a study by Prodam et al. was limited for 2 years which reported that UAG and AG were not predictive of long-term metabolic control in children with type 1 diabetes [45].

Our study has several limitations, the first one being the small number of subjects. Given that we had just 9 patients in each group, our findings should be interpreted with care. Larger numbers of subjects are needed to confirm our study. A second limitation is the lack of control groups without diabetes.

It has been reported that ghrelin levels are age dependent and decrease with age [46]. Because the patients in our study with a higher BMI were older than those with a lower BMI, this could have affected our results. However, as we did not see any differences in AG and UAG levels between overweight and lean type 1 diabetes patient, it is unlikely that age is an important factor in our study.

## Conclusion

To our knowledge, this is the first study to provide data on AG and UAG levels in adults with type 1 diabetes with a diabetes duration of more than 20 years.

Our findings provide some insight into type 1 diabetes by suggesting that the exposure to exogenous insulin seems to be a more important factor in the development of obesity than the ghrelin system. Also the inverse relationship between ghrelin and BMI seems to be lost in type 1 diabetes patients.

## Data Availability

The datasets used and/or analysed during the current study are available from the corresponding author on reasonable request.

## Abbreviations

AEBSF: 4-(2-Aminoethyl) benzenesulfonyl fluoride hydrochloride
AG: Acylated ghrelin
BMI: Body mass index
CV: Coefficient of variation
EIA: Enzyme immunometric assay
GHSR-1a: Growth hormone secretagogue receptor type-1a
GOAT: Ghrelin *O*-acyl-transferase
HbA1c: Glycated hemoglobin
MEC: Medical Ethics Review Committee
STZ: Streptozotocin
UAG: Unacylated ghrelin
